# Characterisation of a human antibody that potentially links cytomegalovirus infection with systemic lupus erythematosus

**DOI:** 10.1038/s41598-019-46329-y

**Published:** 2019-07-10

**Authors:** Jie Ying Jacklyn Neo, Seng Yin Kelly Wee, Isabelle Bonne, Sen Hee Tay, Manfred Raida, Vojislav Jovanovic, Anna-Marie Fairhurst, Jinhua Lu, Brendon J. Hanson, Paul A. MacAry

**Affiliations:** 1 Immunology Programme, Life Sciences Institute, National University of Singapore, Singapore, Singapore; 2 Department of Microbiology & Immunology, Yong Loo Lin School of Medicine, National University of Singapore, Singapore, Singapore; 3 Electron Microscopy Laboratory, Life Sciences Institute, National University of Singapore, Singapore, Singapore; 4 Department of Medicine, National University Health System, Singapore, Singapore; 5 Division of Rheumatology, Department of Medicine, National University Hospital, National University Health System, Singapore, Singapore; 6 Singapore Immunology Network, Agency for Science, Technology and Research (A*STAR), Singapore, Singapore; 7 Singapore Lipidomics Incubator, Life Sciences Institute, National University of Singapore, Singapore, Singapore; 8 Department of Biochemistry, Yong Loo Lin School of Medicine, National University of Singapore, Singapore, Singapore; 9 DSO National Laboratories, Singapore, Singapore

**Keywords:** Antibodies, Autoimmunity, Infection

## Abstract

Human cytomegalovirus (HCMV) is a ubiquitous herpesvirus that has been linked with the development of systemic lupus erythematosus (SLE). Thus far, molecular mimicry has been implicated as the principal mechanism that explains this association. In this study, we characterise a potential alternative process whereby HCMV contributes to SLE. In a cohort of SLE patients, we show a significant association between HCMV infection and SLE through a human antibody response that targets UL44. UL44 is an obligate nuclear-resident, non-structural viral protein vital for HCMV DNA replication. The intracellular nature of this viral protein complicates its targeting by the humoral response – the mechanism remains unresolved. To characterise this response, we present a thorough molecular analysis of the first human monoclonal antibody specific for UL44 derived from a HCMV seropositive donor. This human antibody immunoprecipitates UL44 from HCMV-infected cells together with known nuclear-resident SLE autoantigens – namely, nucleolin, dsDNA and ku70. We also show that UL44 is redistributed to the cell surface during virus-induced apoptosis as part of a complex with these autoantigens. This phenomenon represents a potential mechanism for the bystander presentation of SLE autoantigens to the humoral arm of our immune system under circumstances that favour a break in peripheral tolerance.

## Introduction

The etiology of a pathogenic autoantibody response involves a complex interplay of intrinsic and extrinsic factors that combine to promote immune hypersensitivity. One of the environmental factors implicated in systemic lupus erythematosus (SLE) pathogenesis is human cytomegalovirus (HCMV). The ability of this ubiquitous herpesvirus to establish lifetime latency and periodically shift between lytic and latent stages is thought to evoke and perpetuate SLE reactions. In multiple studies that have demonstrated an association between the HCMV and SLE, the link drawn between the two has been through molecular mimicry^1–3^. In this study, we characterise a potential alternative mechanism through which HCMV can contribute to the humoral response that underlies SLE pathogenesis.

HCMV encodes UL44, a 52 kDa DNA-binding phosphoprotein essential for HCMV DNA replication^4^. Upon translation, UL44 homodimerises and is transported to the nucleus where it interacts with other host and viral antigens to increase HCMV DNA replication efficiency^5^. The interactions of UL44 with host antigens within the nucleus have been described as imperative for HCMV DNA replication^6,7^. However, the nuclear-residency of this viral non-structural protein, makes its targeting by the humoral immune response non-intuitive^8^.

The development of SLE is characterised by the induction and accumulation of autoantibodies against nuclear and cytoplasmic host antigens^9^. It was noted that the progressive accrual of autoantibodies begins up to 9.4 years before the onset of SLE and anti-nuclear antibodies are among the first specificities to emerge. One of the processes thought to contribute to SLE pathogenesis is apoptosis. The induction of apoptosis was observed to trigger the relocalisation and concentration of numerous well-characterised autoantigens, such as SS-A(Ro) and DNA, into apoptotic blebs at the cell surface^10,11^. This results in the exposure of immunologically privileged intracellular self-antigens to humoral immunity^12^. However, apoptosis should render these antigens non-immunogenic^13^. It has been postulated that in susceptible individuals, genetic factors result in a delayed clearance of apoptotic cellular material and this predisposes them to autoimmunity^14^.

In this study, we describe the external display of an obligate intranuclear viral antigen complexed to host factors that have been strongly implicated in SLE. Specifically, we show that the HCMV viral antigen UL44, is redistributed to the plasma membrane as part of a complex that includes nucleolin and dsDNA during virus-induced apoptosis. This potentially explains our observed association between SLE and antibody responses targeting HCMV and UL44. These observations suggest a new potential mechanism where HCMV infection contributes to the development of humoral immune responses against intracellular host antigens.

## Results and Discussion

**Figure 1:**
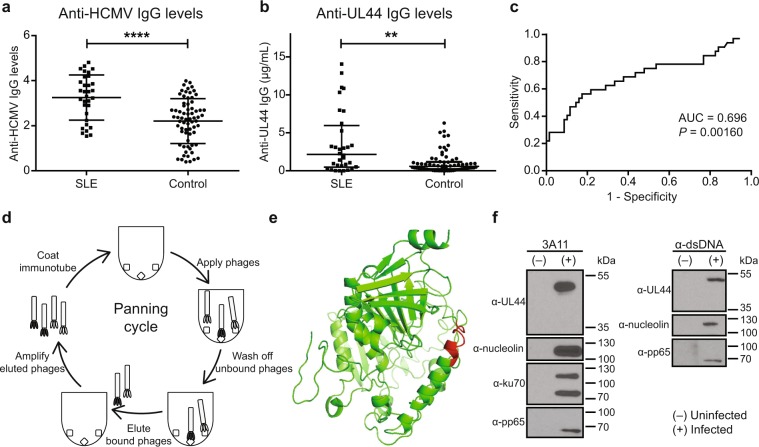
Association between HCMV infection and SLE through UL44 and characterisation of an anti-UL44 antibody. Plasma anti-HCMV and anti-UL44 IgG levels were determined in a cohort of HCMV IgG seropositive 32 SLE patients and 69 controls. (**a**) Plasma anti-HCMV IgG levels were significantly higher in SLE patients than controls (mean of 3.251 vs 2.208; ****P < 0.0001). Results shown are from a representative experiment. The experiment was repeated for a total of 3 times. Data represent mean ± SD. Statistical significance was determined using two-tailed t test. (**b**) Among HCMV(+) individuals with anti-UL44 IgG antibodies, SLE patients had significantly higher plasma concentrations of anti-UL44 IgG antibodies than controls (median of 2.152 vs 0.575 µg/ml; **P = 0.0016). Each data point is an average from 3 independent experiments. Data represent median ± interquartile range. Statistical significance was determined using two-tailed Mann-Whitney U test. (**c**) ROC curve was plotted for anti-UL44 IgG for anti-UL44 IgG(+) SLE and controls (AUC = 0.696; *P* = 0.00160). (**d**) Schematic illustration of the panning process. (**e**) Computational modelling of the HCMV UL44 protein. The epitope targeted by 3A11 is indicated in red. (**f**) Co-immunoprecipitation (Co-IP) was performed using 3A11 (left panel) and anti-dsDNA antibody (right panel) on uninfected (−) or RV1305-infected (+) ARPE-19 cell lysates. Immunoprecipitation products were electrophoresed and immunoblotted separately using monoclonal antibodies against UL44, nucleolin, ku70 and pp65. Co-IP using 3A11 revealed that nucleolin, ku70 and pp65 complexes with UL44. dsDNA was observed to be part of these complexes when co-IP was performed with anti-dsDNA antibody. Full-length blots are presented in Supplementary Fig. 5.

We demonstrate a significant association between HCMV infection and SLE in a cohort of HCMV IgG seropositive individuals. Within our cohort of 32 SLE patients and 69 controls, SLE patients had significantly higher plasma concentrations of anti-HCMV IgG antibodies compared to controls (mean of 3.251 vs 2.208; *P* < 0.0001) (Fig. 1a). This is in concordance with previous studies that show an association between HCMV and SLE^15,16^.

HCMV was previously hypothesised to contribute to SLE pathogenesis through molecular mimicry. The expression of late phase HCMV structural proteins, pp65 and gB, in autoimmune-prone mice induced the expression of antibodies that cross-react with autoantigens^1,17^. With the knowledge that UL44, an early phase protein, is one of the most immunogenic HCMV proteins, we similarly tested plasma anti-UL44 IgG concentration^18^. We observed that SLE patients had significantly higher plasma concentrations of anti-UL44 IgG antibodies than controls (median of 2.152 vs 0.575 µg/mL; *P* = 0.0016) (Fig. 1b). An ROC analysis was conducted to determine the predictive potential of measuring anti-UL44 IgG antibodies in SLE (AUC = 0.696; *P* = 0.00160) (Fig. 1c). Based on the computed AUC, SLE patients have a 69.6% chance of having a higher anti-UL44 IgG concentration than controls. As a predictive marker, anti-UL44 IgG had a specificity of 85.5% at a sensitivity of 90.6%. This is comparable to that of dsDNA, 60 kDa SSA and complement C4d with AUC values of 0.74, 0.72 and 0.64 respectively^19–21^. However, we did not observe a correlation between anti-UL44 IgG concentrations and SLE activity as determined by SLEDAI (Supplementary Fig. 1).

Given the observed link between UL44 and SLE, we sought to develop a human monoclonal antibody (mAb) against UL44 that will allow us to further study this association. An immune antibody phage display library was created from an individual with a high titer of anti-HCMV antibodies. Repeated rounds of panning the library against HCMV antigens resulted in the isolation and expression of 3A11, a fully-human anti-UL44 mAb (Fig. 1d and Supplementary Fig. 2). 3A11 was engineered as a human IgG1 and was found to have binding activity for the HCMV antigen but not neutralising activity for the whole virus (Supplementary Fig. 2). A kinetic analysis of UL44 expression in HCMV-infected cells was conducted. We observed expression of UL44 from 24 h post infection, confirming that it is a delayed early antigen (Supplementary Fig. 2). Epitope mapping of 3A11 was performed and we show that the binding interface is part of an alpha-helical structure on an exposed domain that was previously proposed to be immunodominant (Fig. 1e)^18,22^.

UL44 is a DNA-binding protein essential for HCMV DNA replication. To analyse the interaction partners of UL44, co-immunoprecipitation was performed using 3A11 and an anti-dsDNA antibody. Results confirmed that UL44 exists as a complex with the HCMV antigen pp65 and autoantigens such as nucleolin, dsDNA and ku70 (Fig. 1f and Supplementary Fig. 5). The interaction between UL44 and autoantigens, nucleolin and ku70, was noted as early as 24 h post infection in the delayed early phase of infection (Supplementary Fig. 3).

**Figure 2:**
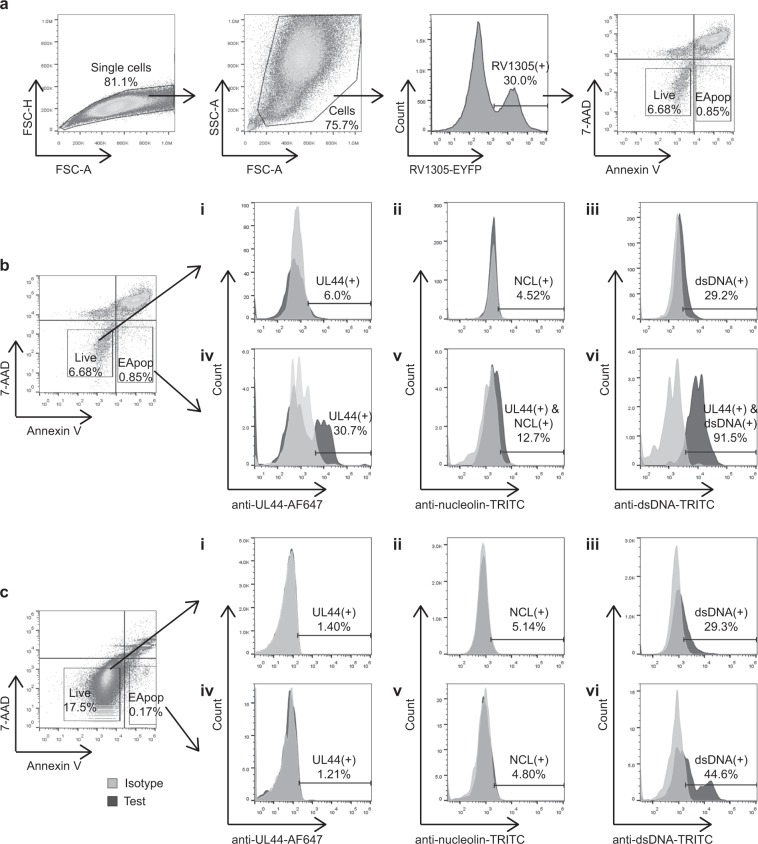
Expression of UL44 and interacting host antigens on ARPE-19 cell surface. **(a)** Gating strategy for infected APRE-19 cells. ARPE-19 cells were infected with RV1305, a strain of HCMV that expresses an EYFP fusion protein. Cells were stained with 7-AAD and annexin V before flow cytometric analysis. Live cells stain 7-AAD(−), annexin V(−) while early apoptotic (EApop) cells stain 7-AAD(−), annexin V(+). The gating strategy for uninfected cells was the same except that the cells were not gated based on EYFP expression before live/dead gating. **(b)** Flow cytometric analysis of RV1305-infected ARPE-19 cells post surface staining. Relative to isotype control antibody-stained cells, UL44 was observed on 30.7% of early apoptotic cells (iv), but not on live cells (i). Gating on UL44(+) cells revealed that 12.7% and 91.5% of the apoptotic cells displayed nucleolin (NCL) (v) and dsDNA (vi) respectively. In contrast, only 4.52% and 29.2% of the live cells displayed NCL (ii) and dsDNA (iii) respectively. **(c)** Flow cytometric analysis of uninfected ARPE-19 cells was performed to check for the specificity of 3A11 (i and iv) and surface expression of nucleolin (ii and v) and dsDNA (iii and vi). Nucleolin expression was not observed on uninfected cells. Relative to the isotype control, 29.3% of live (iii) and 44.6% of early apoptotic (vi) ARPE-19 cells stained positive for dsDNA.

For B cells to be activated to produce antibodies against specific antigens, their targets need to be accessible to the extracellular immune milieu. However, UL44 is known to be localised intracellularly inside the nucleus of HCMV-infected cells^23,24^. To understand how UL44 became an immunodominant target, surface staining of HCMV-infected ARPE-19 cells was performed using 3A11 (Fig. 2). Flow cytometric analyses revealed that UL44 is detected on the surface of a subpopulation of early apoptotic [7AAD(−), annexin V(+)] cells. UL44 was not detected on the surface of infected, live [7AAD(−), annexin V(−) cells (Fig. 2b,i,iv).

We hypothesised that the translocation of UL44 to the cell surface during apoptosis would result in a concomitant presentation of its host interaction partners. Surface staining of HCMV-infected cells was performed using 3A11 and anti-nucleolin or anti-dsDNA antibodies. Gating on UL44(+) cells revealed an increased surface expression of nucleolin and dsDNA on early apoptotic cells compared to live cells (Fig. 2b,v,vi). To determine whether the observed differences were due to HCMV infection and/or apoptosis, uninfected cells were similarly stained for these antigens (Fig. 2c). In both uninfected and infected cells, proportions of live cells exhibiting surface expression of nucleolin and dsDNA were similar. This indicated that HCMV infection did not effect a change in the surface expression of nucleolin and dsDNA in live cells. However, proportions of apoptotic cells expressing nucleolin and dsDNA were lower in uninfected than UL44-expressing infected cells. Taken together, these results indicate that in apoptotic cells, surface expression of nucleolin and dsDNA increased as a result of the expression of UL44.

**Figure 3:**
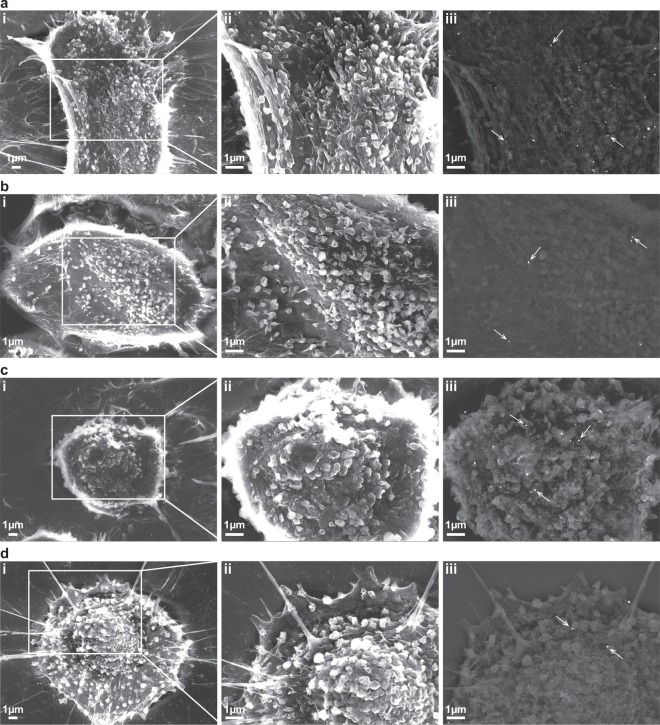
Scanning electron microscopy analyses of infected ARPE-19 cells stained with 3A11. Representative images of RV1305-infected ARPE-19 cells stained with either 3A11 **(a**,**c)** or an isotype control antibody **(b**,**d)**, and counterstained with an anti-human IgG-15 nm nanogold conjugate antibody. Nanogold particles are indicated with white arrows. Number of nanoparticles was significantly higher on 3A11- than isotype antibody-stained cells (median of 16 vs 6.5; ****P < 0.0001) (Supplementary Fig. 4). Cells from two different phases of apoptosis, based on cellular morphology, are shown. Cells from earlier phases of apoptosis **(a**,**b)** appear bigger and flatter than those in later stages **(c**,**d)**. Results revealed that 3A11 stained more intensely in areas of cells undergoing apoptotic blebbing. Images were acquired using the JEOL JSM 6701F field emission scanning electron microscope operating at 10 kV. In all panels, (i) and (ii) are secondary electron images taken at 5,000x and 10,000x magnifications respectively; (iii) are backscattered electron images taken at 10,000x magnification.

To better visualise the surface expression of UL44 in HCMV-infected cells, we performed immunogold labelling followed by scanning electron microscopy. The specificity of 3A11 staining was confirmed by the greater number of nanogold particles on 3A11-stained cells relative to that with isotype antibody control (Supplementary Fig. 4). A morphological examination of the cell surface revealed that UL44 was concentrated proximal to sites of membrane blebbing. Cell surfaces that were relatively intact stained with fewer nanogold particles (Fig. 3). The increased staining by 3A11 around apoptotic blebs suggests that the translocation of UL44 from the nucleus to cell surface was linked to apoptosis.

HCMV infection is proposed to be clinically inconsequential in immunocompetent individuals. However, in transplant recipients, HCMV infection is associated with the induction of autoantibodies, potentially contributing to allograft rejection^25^. In this study, we demonstrate that UL44, a nucleus-localised HCMV antigen can be detected on the cell surface during apoptosis. We also show the externalisation of this immunogenic HCMV protein is accompanied by interacting host antigens, nucleolin and dsDNA. The relocalisation of intracellular self-antigens to the cell surface, triggered by apoptosis, has been implicated in SLE pathogenesis^26,27^. However, apoptosis is an inherently quiescent and non-inflammatory process^13^. We propose that the concomitant presentation of an immunogenic viral protein directly linked to host nuclear proteins on the surface of HCMV-infected cells potentially adjuvantises the self-antigens, contributing towards a break in peripheral tolerance.

As mentioned, HCMV has been previously linked to SLE through alternative mechanisms. One of the ways that microbial infections are commonly thought to contribute to autoimmunity is through molecular mimicry. In the case of HCMV, antibodies developed *in vivo* in mice against tegument protein pp65 and structural protein gB have been observed to cross-react with multiple host antigens such as the U1-70 kDa spliceosome protein and dsDNA^1,3,17^. More recently, Guo *et al*. observed that HCMV US31 protein interacts with NF-κB2 to induce inflammation in mono-macrophages, perturbs the innate immune system to impair immune homeostasis and eventually promote SLE development^28^. The observations in our study suggest a different mechanism, through the humoral immune response, that HCMV can potentially contribute to the development of the autoantibody response that is characteristic of SLE.

Despite central tolerance, it is estimated that up to 20% of peripheral mature naïve B cells are specific for nuclear antigens in healthy individuals^29^. This has been linked to the intracellular sequestration of these autoantigens that impacts upon the efficiency of clonal deletion in the bone marrow^30^. Given the existence of potentially self-reactive B cells in the peripheral circulation, an important checkpoint for the induction of autoantibodies is the activation of autoreactive CD4^+^ helper T cells. Sanderson *et al*. demonstrated *in vitro* that the co-capture of unlinked host and viral proteins – host myelin oligodendrocyte glycoprotein and influenza hemagglutinin – by B cells resulted in the induction of autoimmunity when (i) anti-viral B cells activate autoreactive T cells and/or (ii) autoimmune B cells recognising the displayed host epitopes are activated by anti-viral T cells^31^. Taken together, our study highlights a mechanism where complexed, nucleus-restricted virus and host antigens can potentially contribute to humoral responses that underlie autoimmunity.

UL44 is a phosphoprotein containing nuclear localisation signals with no transmembrane domain(s). Its function as a DNA processivity factor is restricted to the nucleus during DNA replication. A possible explanation to our detection of UL44 on the plasma membrane is that its translocation to the surface during apoptosis is a result of its interaction with host nuclear antigens.

It is unclear whether HCMV infection occurred before or after the onset of SLE disease in this patient cohort and thus we could not make a definitive conclusion about a causal link between the concomitant expression of UL44 with SLE autoantigens and the development of an autoantibody response. Taking into account the strict species specificity of cytomegaloviruses, studies *in vivo* in mice would need to utilise the murine counterpart of HCMV. To determine the link between HCMV infection and SLE, the effect of cytomegalovirus infection can be explored in SLE-prone MRL/lpr and NZB/NZW F1 mice. It was observed that these mice develop autoantibodies against self-antigens such as nucleolin, histones and dsDNA naturally by 4 weeks of age^32^. The serostatus of the mice can be followed post murine CMV infection, to determine if the infection hastens the development of anti-nuclear antibodies.

To explore the mechanistic link between UL44 and SLE, the expression of HCMV UL44 can be induced in SLE-prone MRL/lpr mice through the use of an expression plasmid encoding HCMV UL44. There is a 78% identity between the amino acid sequences of the human and murine nucleolin proteins. Co-immunoprecipitation experiments can be performed to determine the presence of the interaction between HCMV UL44 and murine nucleolin, ku70 and dsDNA. The induction of autoantibodies against the complexed autoantigens and the subsequent development of SLE phenotypes will suggest the potential for HCMV UL44 to induce autoimmunity *in vivo*.

In summary, this study suggests a potential mechanism where HCMV infection can potentiate the development of autoantibodies against nuclear proteins that are strongly associated with SLE. We observe for the first time the externalisation of UL44, a HCMV DNA-binding nuclear protein, complexed to autoantigens nucleolin and dsDNA during apoptosis. Taken together with the observed significantly higher anti-UL44 IgG antibody levels in SLE patients, these observations suggest a new alternative pathway through which HCMV can contribute to SLE development. This phenomenon and the future elucidation of the mechanistic link will provide valuable insights that should inform HCMV vaccine design and SLE diagnosis decisions.

## Methods

### Study approval

The study was conducted according to the principles of the Declaration of Helsinki. Human peripheral blood was obtained with written informed consent (NHG DSRB Ref: DSRB-B/07/195, 2014/00600 and 2016/01419). The study protocol was approved by the National Healthcare Group (NHG).

### Cell line and maintenance

ARPE-19 cell line was obtained from ATCC (CRL-2302) and cultured in DMEM/F12, HEPES supplemented with 10% FBS. Media used in virus culture and assays was supplemented with 2% FBS instead.

### ELISA to determine plasma anti-HCMV IgG concentration

RV1305 was propagated in ARPE-19 cells for two weeks. Culture medium was harvested and clarified at 12,000 g, 10 minutes before concentration at 40,000 g, 2 hours. Concentrated virus was resuspended in PBS for use in ELISA. Maxisorp 96-well plates were coated with 50 µL/well of virus, overnight at 4 °C. The plates were washed with PBS, blocked using 4% skim milk before incubation with plasma diluted 1:250 in 2% skim milk. 50 µL/well of the diluted plasma was added in duplicate and incubated for 1.5 hours, room temperature. Plates were washed and incubated with goat anti-human IgG Fc secondary antibody, HRP (#31413, Invitrogen), diluted to 1:3000 in 2% skim milk. Signals were developed with 1-Step Ultra TMB-ELISA Substrate and quenched with 1 M sulphuric acid. Absorbance was measured at 450 nm. Rabbit anti-CMV polyclonal antibody (ab21015, Abcam) was used as a positive control. Plasma from a clinically-tested HCMV IgG seronegative individual was used as a negative control. Absorbance values obtained were divided by that from the positive control. To determine the threshold above which an individual would be classified as anti-HCMV IgG seropositive, ratios were compared to that of the negative control. Individuals with ratios more than twice that of the negative control were classified as seropositive.

### ELISA to determine plasma anti-UL44 IgG concentration

The full-length UL44 protein was expressed in *Escherichia coli* strain, BL21. The protein was recovered from the insoluble fraction of the bacterial lysate and dialysed to 2 M urea for use in ELISA. Maxisorp 96-well plates were coated with 5 µg/mL (50 µL/well) of mouse anti-UL44 monoclonal antibody (P1202, Virusys) overnight at 4 °C. Plates were then washed with PBS and blocked using 4% skim milk. Plates were incubated with 8 µg/mL (50 µL/well) of UL44. Plates were washed twice with PBS/0.5%Tween and once with PBS. Plasma samples were diluted 1:500 in 2% skim milk. 50 µL/well of the diluted plasma was added in duplicate and incubated for 1.5 hours at room temperature. The reaction was developed as described above. To determine the concentration of anti-UL44 IgG in the plasma, signals obtained from plasma samples were compared against a standard curve constructed using 3A11 at various concentrations.

### Immune antibody phage library creation and biopanning

Peripheral blood mononuclear cells were isolated from donor blood using Ficoll density gradient centrifugation. B cell purification was performed by negative selection with a B cell enrichment kit (STEMCELL Technologies). Using the method described previously, the antibody phage library was created to display Fab fragments on the phage pIII protein^33^. The library was screened for HCMV-specific phages by selection on concentrated HCMV. Selected phages were converted to human IgG1 antibodies. Recombinant antibodies were expressed in HEK293-6E cells and purified using Protein G sepharose beads.

### Epitope mapping using ELISA

The UL44 peptide library used comprised 15-mer biotinylated peptides with 10-mer overlaps (Mimotopes). Lyophilised peptides were reconstituted in 200 µL 80% DMSO/20% water to create a stock solution of each peptide. Streptavidin-coated plates were used for epitope mapping. Peptides were diluted to a working strength of 1/1000 in 0.1% BSA/0.1% sodium azide/PBS. 100 µL of each peptide was added to the streptavidin-coated plates and incubated at room temperature for 1 hour. The plate was then washed 4 times in PBS/0.1% Tween-20 (PBS/0.1%T). 100 µL of the antibody-of-interest, diluted to 5 µg/mL in 2% BSA/PBS, was then added to each well and incubated for 1 hour at room temperature. The plate was washed as before. 100 µL/well of goat anti-human IgG Fc secondary antibody, HRP, diluted to 1:3000 in 2% BSA/PBS (#31413, Invitrogen) was added and incubated for 1 hour at room temperature. The plate was washed and detected as before. Computational modelling of the full-length UL44 protein structure was performed using I-TASSER^22^.

### Co-immunoprecipitation

ARPE-19 cells were infected with RV1305 at a multiplicity of infection of 0.1. When the cells exhibited 100% cytopathic effect, the monolayer was gently washed with ice-cold PBS. Co-immunoprecipitation was performed as previously described^6^. To prepare the cell lysates for use in immunoprecipitation, the following lysis buffer was used: 50 mM HEPES [pH 7.4], 1% Triton X-100, 150 mM sodium chloride, 10% glycerol, 0.1 mM dithiothreitol, 2 mM EDTA). All buffers used were supplemented with protease inhibitor. Clarified cell lysate was first incubated with 5 µg of isotype antibody, overnight at 4 °C. Equilibrated Protein G sepharose beads were then added and allowed to incubate on a rotator, for 2 hours at 4 °C. After centrifugation to remove the beads, the supernatant was equally divided and incubated with 5 µg of antibody-of-interest, isotype antibody or PBS, overnight at 4 °C. Equilibrated Protein G sepharose beads were then added to the samples and incubated on a rotator, for 2 hours at 4 °C. Beads were spun down and the supernatant was removed. The beads were washed thrice in lysis buffer diluted in PBS. After the last wash, the supernatant was removed and SDS-PAGE loading buffer was added, boiled and used for SDS-PAGE and western blotting. For immunoblotting, the following antibodies recognising UL44 (CA006-100, Virusys), nucleolin (ab13541, Abcam), dsDNA (ab27156, Abcam), ku70 (ab201963, Abcam), IE1 (C9100-18, US Biological) and HCMV pp65 (ab43041, Abcam) were employed.

### Flow cytometry

ARPE-19 cells were infected with RV1305 at a multiplicity of infection of 0.8 and used for flow cytometry. Uninfected cells were included as negative controls. Before staining, cell monolayers were rinsed twice with pre-warmed Dulbecco’s phosphate-buffered saline (DPBS) and detached using pre-warmed 1X TrypLE (Thermo Fisher Scientific) at 37 °C. Detached cells were resuspended in staining buffer (DPBS/2% rabbit (Rb) serum/0.09% sodium azide) with a total of 10% Rb serum. Cells were pelleted and blocked in 10% Rb serum in staining buffer on ice for 30 minutes. Cells were then pelleted and resuspended in staining buffer. For each test, 10^6^ cells were stained with 10 µg/mL primary antibodies, for 30 minutes on ice. Cells were then washed and stained with the relevant secondary antibodies, 15 minutes on ice. Secondary antibodies used include: Goat anti-Human IgG Secondary Antibody/Alexa Fluor 647 (A21445, Invitrogen) and F(ab′)2-Donkey anti-Mouse IgG Secondary Antibody/TRITC (A24511, Invitrogen). Cells were washed twice in annexin V staining buffer before live/dead staining was performed. For live/dead staining, cells were resuspended in 7-AAD (Invitrogen) at 10 µg/mL and 5 µL/test of annexin V, PB conjugate (Invitrogen). The cells were left to incubate for 15 minutes at room temperature before acquisition on the Attune NxT flow cytometer (Thermo Fisher Scientific) and analysis using FlowJo version 10.

### Scanning electron microscopy

RV1305-infected ARPE-19 cells were maintained on poly-L-lysine-coated glass cover slips in 24-well prior to immunostaining. Before staining, cells were washed once with PBS followed by blocking with blocking buffer (10% Rb serum/0.09% sodium azide/PBS) on ice for 30 minutes. Cells were then stained with 50 µL of either 3A11 or its isotype antibody at 10 µg/mL, for 30 minutes at room temperature. Cover slips were washed with PBS before fixing with 4% paraformaldehyde at room temperature for 10 minutes. Paraformaldehyde was then quenched with 10 mM glycine/PBS for at least 5 minutes at room temperature. Cover slips were rinsed once in PBS before blocking in 10% BSA/PBS. Cells were then stained with goat anti-human IgG–15 nm nanogold conjugate (#25213, Electron Microscopy Sciences) diluted 1:20 in 1% BSA/PBS for 30 minutes at room temperature.

To treat the cells for scanning electron microscopy viewing, cover slips were rinsed once in PBS before fixing with 1% glutaraldehyde for 10 minutes at room temperature. After fixing, cells were washed with 50 mM glycine followed by 1% BSA/PBS and then in distilled water. The nanogold particles were chemically enhanced using GoldEnhance EM Plus (Nanoprobes) for 10 minutes and rinsed in distilled water. Cells were fixed with 1% osmium tetraoxide/PBS for 45 minutes at room temperature. As before, cells were washed thrice with PBS, 5 minutes per wash. Cells were then dehydrated in a graded series of ethanol, for 10 minutes in each concentration. Starting at 30% ethanol, cells were progressively dehydrated in 50%, 70%, 90% and 100% ethanol. Samples were then dried using the critical point drying method. Dried samples were sputter coated with carbon, using a LEICA EM ACE200 evaporator. Samples were examined and photographed with a JEOL JSM 6701F field emission scanning electron microscope operating at 10 kV.

### Statistical analysis

Statistical data were generated with GraphPad Prism version 5.04. Normality was tested using the Shapiro-Wilk normality test. Where data follow normal distribution, two-tailed t-test was used. Two-tailed Mann-Whitney U test was used when data was not normally distributed. *P* ≤ 0.05 was considered to be statistically significant.

## Supplementary information

Supplementary Information

## Data Availability

All data generated or analysed during this study are included in this published article and its Supplementary Information files. Extra data are available from the corresponding author upon request.
