# Supplementary material for: Characterisation of a human antibody that potentially links cytomegalovirus infection with systemic lupus erythematosus
Source: Sci Rep. 2019 Jul 10;9:9998. doi: 10.1038/s41598-019-46329-y (PMC6620320; doi:10.1038/s41598-019-46329-y)
Supplement: Supplementary file 1 — Supplementary Information [file 41598_2019_46329_MOESM1_ESM.pdf]

Supplementary material for the manuscript:

Characterisation of a human antibody that potentially links cytomegalovirus infection with systemic lupus erythematosus

Jie Ying Jacklyn Neo[1,2], Seng Yin Kelly Wee[1,2], Isabelle Bonne[3], Sen Hee Tay[2,4-6], Manfred Raida[7,8], Vojislav Jovanovic[1], Anna-Marie Fairhurst[2,6], Jinhua Lu[1,2], Brendon J. Hanson[9] & Paul A. MacAry[1,2,*]

[1]Immunology Programme, Life Sciences Institute, National University of Singapore, Singapore.

[2]Department of Microbiology & Immunology, Yong Loo Lin School of Medicine, National University of Singapore, Singapore.

[3]Electron Microscopy Laboratory, Life Sciences Institute, National University of Singapore, Singapore.

[4]Department of Medicine, National University Health System, Singapore.

[5]Division of Rheumatology, Department of Medicine, National University Hospital, National University Health System, Singapore.

[6]Singapore Immunology Network, Agency for Science, Technology and Research (A*STAR), Singapore.

[7]Singapore Lipidomics Incubator, Life Sciences Institute, National University of Singapore, Singapore.

[8]Department of Biochemistry, Yong Loo Lin School of Medicine, National University of Singapore, Singapore.

[9]DSO National Laboratories, Singapore.

*Correspondence and requests for materials should be addressed to P.A.M. (email: micpam@nus.edu.sg)

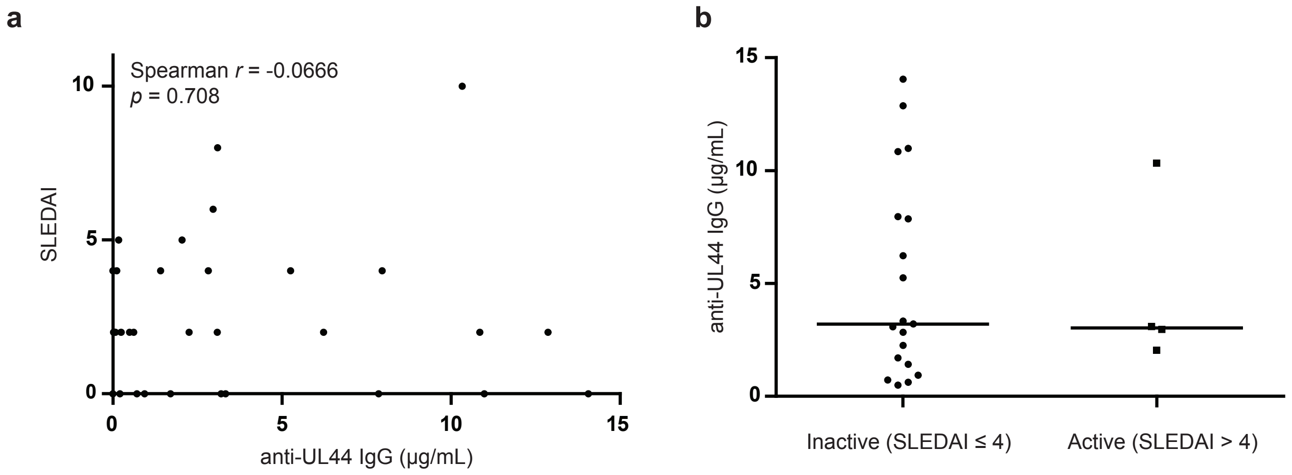

Supplementary Figure 1. Association between anti-UL44 IgG levels and SLEDAI. The following analyses were performed for SLE patients whose information was available. **(a)** Correlation between SLEDAI and plasma anti-UL44 IgG concentration (Spearman r = -0.0666; p = 0.708). **(b)** SLE patients were grouped based on their SLEDAI scores. Patients with SLEDAI > 4 were considered to be undergoing active SLE. There was no significant difference between the plasma anti-UL44 IgG concentrations of patients with inactive and active SLE (P = 0.968, two-tailed Mann-Whitney U test).

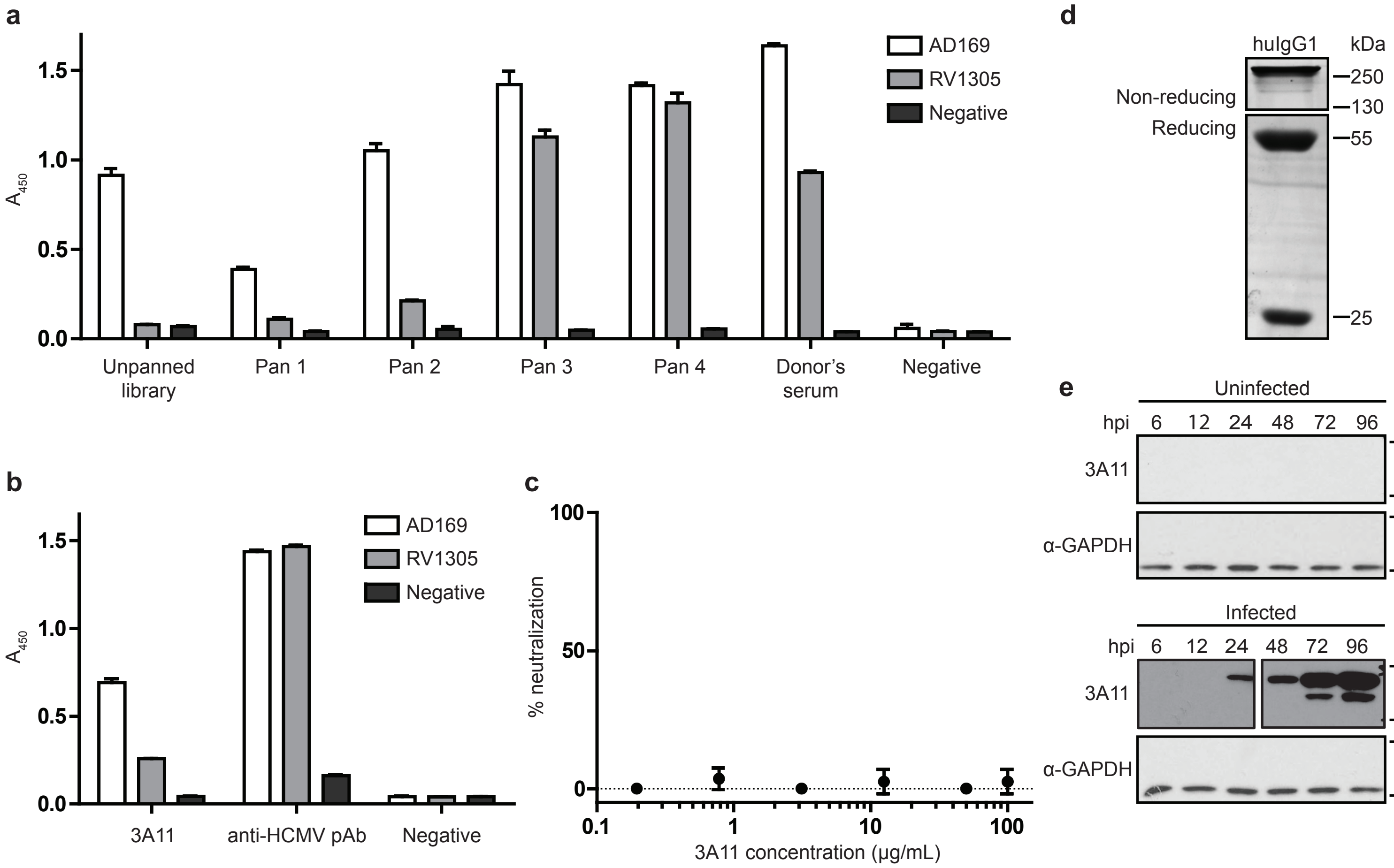

Supplementary Figure 2. Discovery and characterisation of 3A11, an anti-UL44 antibody. (a) ELISA results using polyclonal phages obtained from each round of panning. **(b)** ELISA results using the expressed human IgG1 3A11. **(c)** Foci reduction neutralisation test using 3A11. Data represent mean ± SD. **(d)** Purified 3A11 electrophoresed under reducing and non-reducing conditions on SDS-PAGE gel. Uncropped gel image is presented in Supplementary Figure 6(a). **(e)** Western blot using 3A11 on uninfected and infected ARPE-19 cell lysates harvested at different time points post infection. In blot using infected cell lysate immunostained with 3A11, image from 48 to 96hpi was obtained at a shorter exposure than that from 6 to 24hpi. Full-length blots are presented in Supplementary Figure 6(b-d). (hpi: hours post-infection)

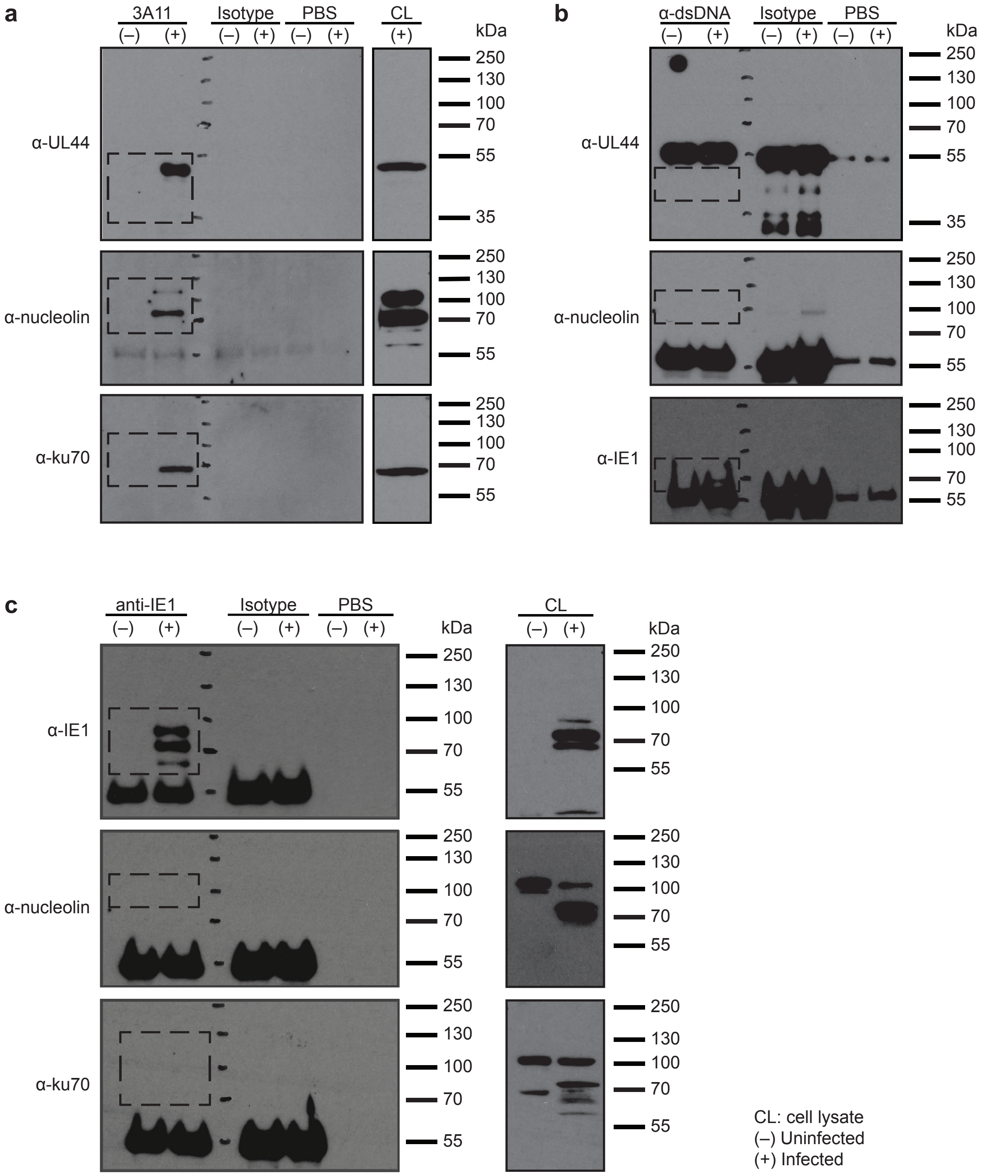

Supplementary Figure 3. Co-immunoprecipitation using uninfected or RV1305-infected ARPE19 cell lysates harvested at 24h post infection. (a) Co-IP using 3A11 revealed that the autoantigens-of-interest, nucleolin and ku70, were complexed with UL44 even in the delayed early phase of infection, at 24 hours post infection (hpi). **(b)** Co-IP using anti-dsDNA antibody showed that at 24hpi, UL44 and nucleolin could not be detected as components of a complex with dsDNA. HCMV intermediate early 1 (IE1) protein was detected as a ~70 kDa band in the complex immunoprecipitated by anti-dsDNA. **(c)** Co-IP was performed using an antibody that targets the IE1 protein (left column). Results revealed that nucleolin and ku70 were not part of a complex with IE1 protein. Immunoblotting was similarly performed on the cell lysates used for the co-IP experiment to confirm the presence of the proteins-of-interest in the harvested cell lysates used for co-IP (right column).
The dashed boxes indicate the regions of interest on the blots. All blots presented in this figure are full-length blots.

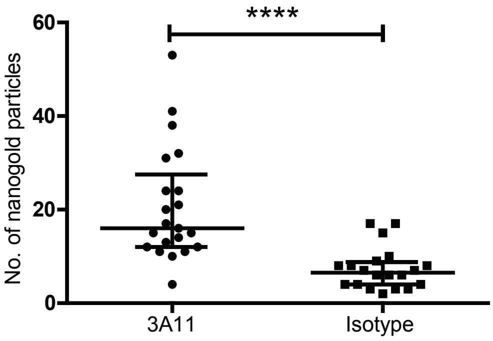

Supplementary Figure 4. Enumeration of nanoparticles on infected ARPE-19 cells stained with 3A11 and an isotype control antibody. RV1305-infected ARPE-19 cells were stained with either 3A11 or an isotype control antibody, and counterstained with an anti-human IgG-15nm nanogold conjugate antibody. Nanoparticles were enumerated on both 3A11- (n = 21) and isotype antibody- (n = 20) stained RV1305-infected ARPE-19 cells. Number of nanoparticles was significantly higher on 3A11- than isotype antibody-stained cells (median of 16 vs 6.5; ****$P < 0.0001$). Data represent median ± interquartile range. Statistical significance was determined using two-tailed Mann-Whitney U test.

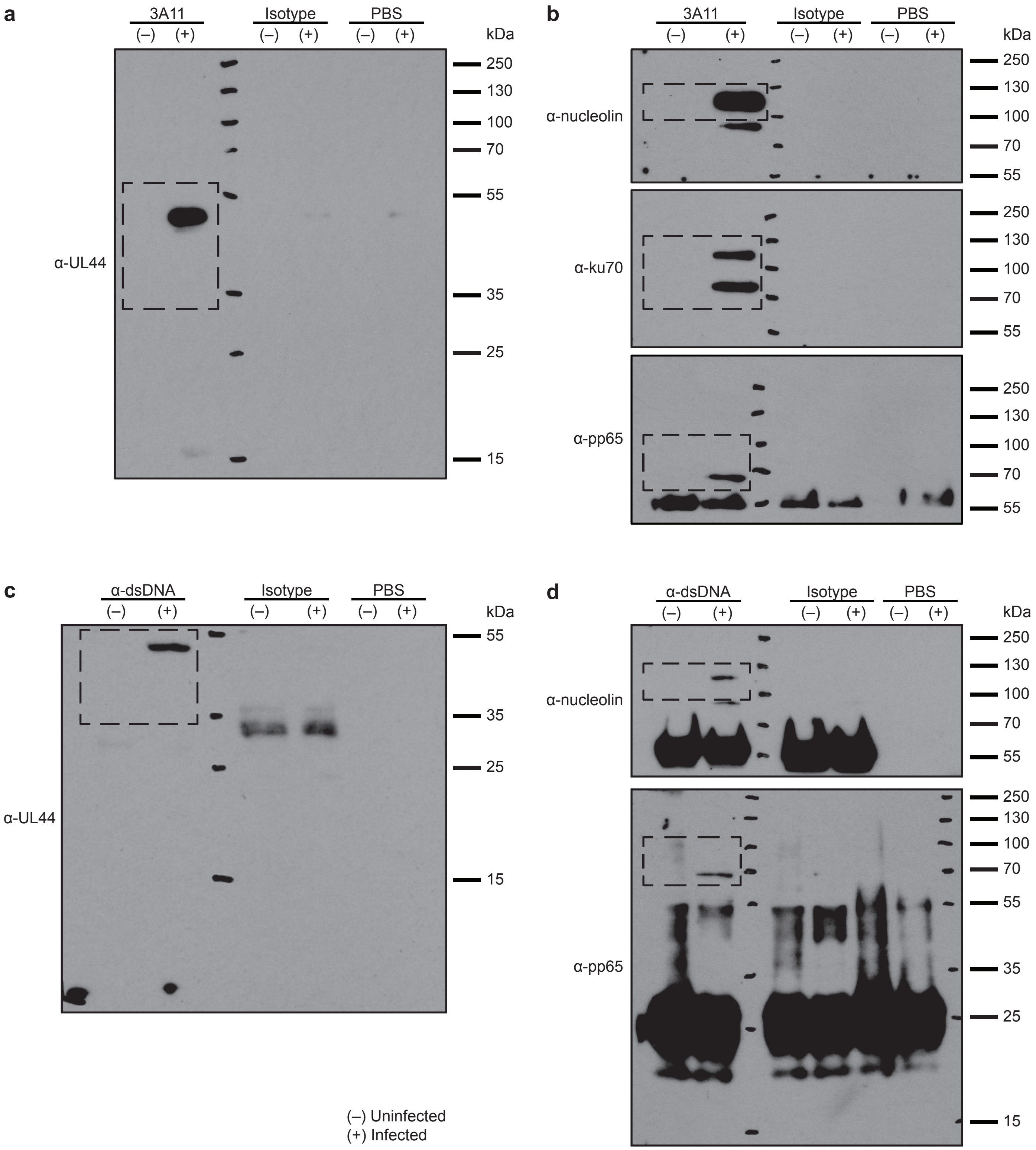

Supplementary Figure 5. Accompanying full-length blots to Figure 1(f). (a and b) Accompanying blots to the left panel of Figure 1(f). Co-immunoprecipitation was performed using 3A11. The immunoprecipitated products were electrophoresed and immunoblotted separately using monoclonal antibodies against UL44, nucleolin, ku70 and pp65. **(c and d)** Accompanying blots to the right panel of Figure 1(f). Co-immunoprecipitation was performed using anti-dsDNA antibody. The immunoprecipitated products were electrophoresed and immunoblotted separately using monoclonal antibodies against UL44, nucleolin and pp65. Sections used for Figure 1(f) are indicated by the dashed boxes.

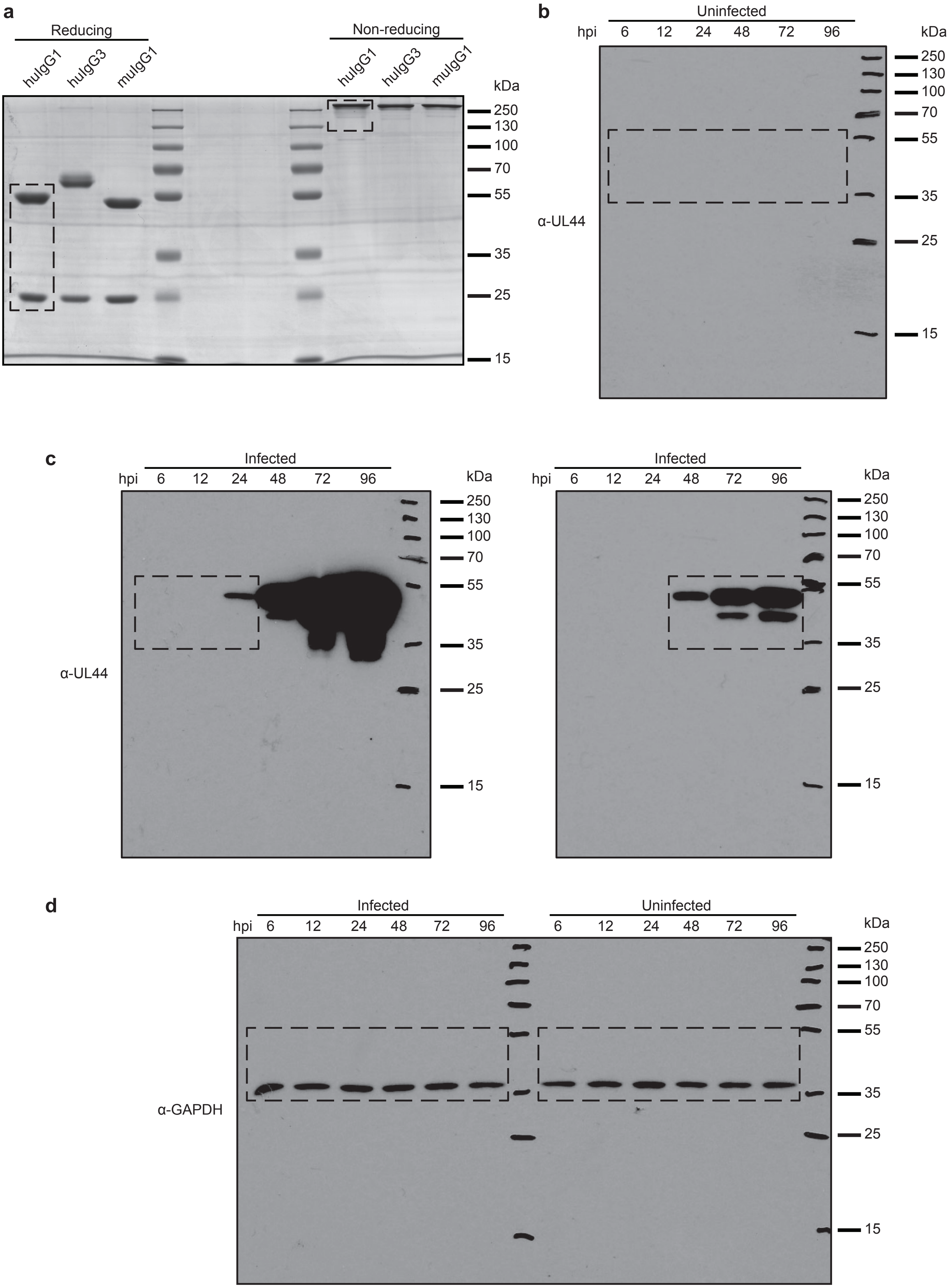

Supplementary Figure 6. Accompanying full-length blots to Supplementary Figure 2(d) and (e). (a) The accompanying coomassie-stained SDS-PAGE gel photo to Supplementary Figure 2(d). **(b-d)** The accompanying western blot film images to Supplementary Figure 2(e). The left panel in (c) was taken at a longer exposure than that on the right. Areas used are indicated by the dashed boxes. (hpi: hours post-infection)

Supplementary Table S1. Data for cohort used in statistical analysis

	SLE patients	Controls	Statistical test (p-value)
**Age**			0.0284[1]
21-30	8	29	N.S.
31-40	11	21	N.S.
41-50	5	6	N.S.
51-60	5	4	N.S.
61-70	2	–	–
Data not available	1	9	–
**Sex**			N.S.[2]
Male	6	19	
Female	26	41	
Data not available	–	9	
**Smoking status**			–
Current smoker	3	1	
Ex-smoker	4	–	
Non-smoker	10	34	
Data not available	15	34	
**Alcohol**			–
Non-drinker	12	2	
Ex-drinker	1	–	
Data not available	19	67	

[1]two-tailed Mann Whitney U test; [2]Fisher's exact test; N.S.: Not significant

Supplementary Table S2. Comparison of anti-HCMV IgG levels within the different age groups

	SLE patients	Controls	Statistical test (p-value)*
**Age**	36 (28-43)	31 (26.25-37)	0.0284
21-30	3.54 (3.25-3.90)	2.34 (1.11-2.69)	0.0006
31-40	2.92 (1.67-3.55)	2.69 (1.48-3.32)	N.S.
41-50	2.05 (1.84-4.49)	2.02 (1.44-3.59)	N.S.
51-60	4.37 (2.82-4.59)	2.17 (2.0-2.48)	0.0159
61-70		N.A.	

* two-tailed Mann Whitney U test
